# Supplementary figures and images for: Morphology of inner cell mass: a better predictive biomarker of blastocyst viability
Source: PeerJ. 2022 Aug 26;10:e13935. doi: 10.7717/peerj.13935 (PMC9422976; doi:10.7717/peerj.13935)

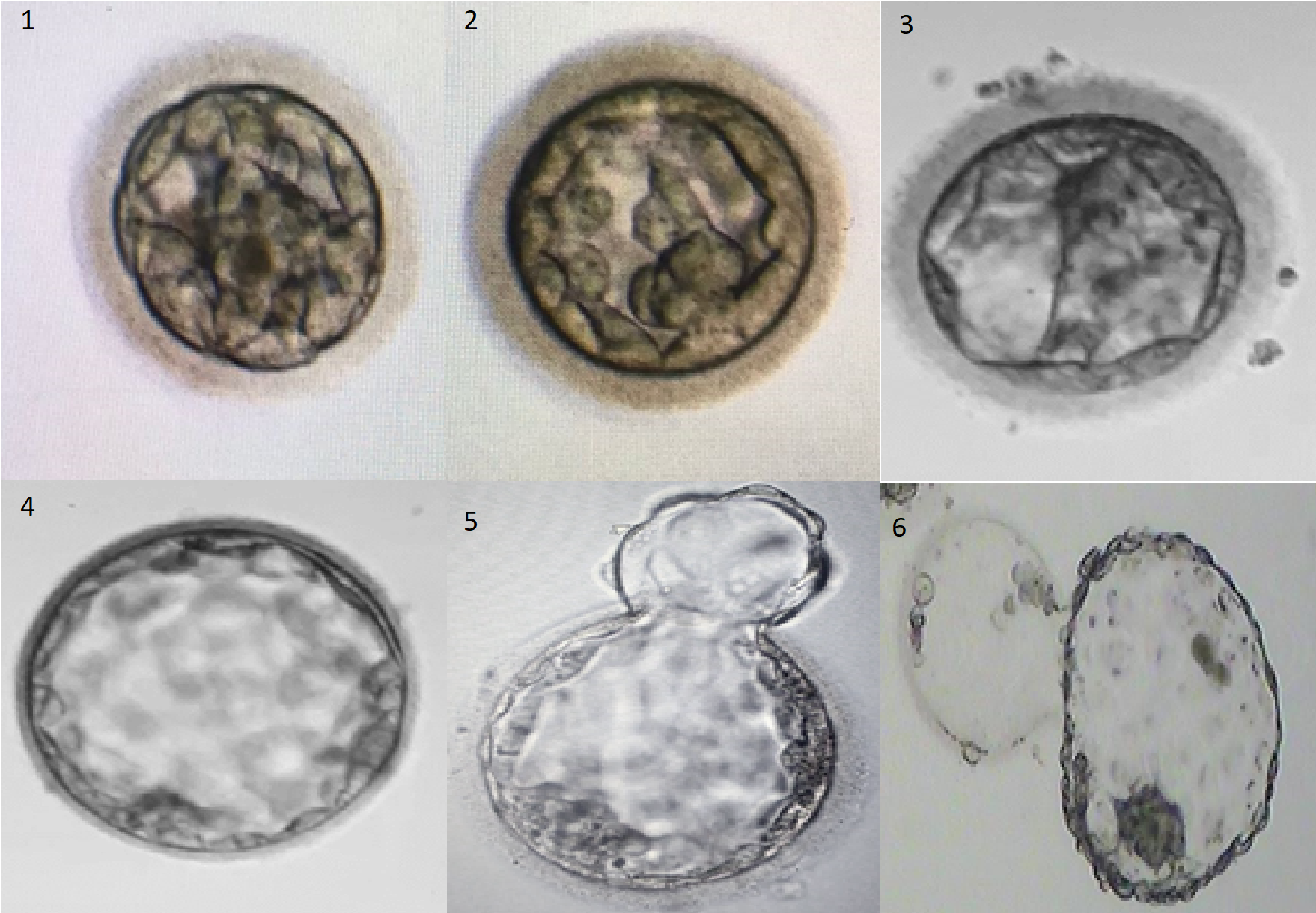

Supplement: Figure S1 — Each picture indicates the blastocyst grade based on the degree of expansion proposed by Gardner.and Schoolcraft. (A) 1- Early Blastocyst (B) 2- Blastocyst (C) 3- Expanding Blastocyst (D) 4 - Expanded Blastocyst (E) 5 - Hatching Blastocyst (F) 6 - Hatched Blastocyst [file peerj-10-13935-s002.png]

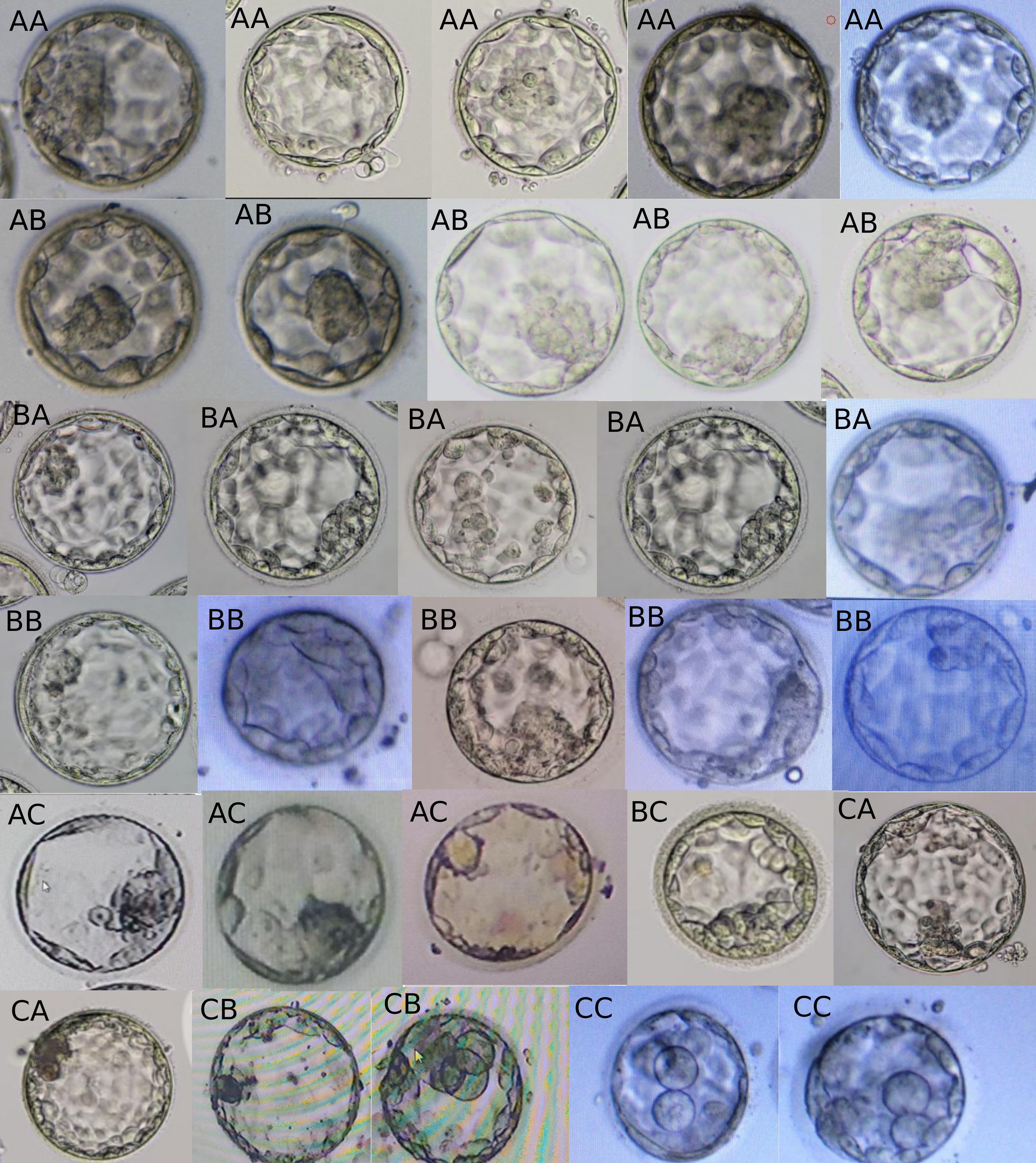

Supplement: Figure S2 — Different grades of blastocysts with possible combinations of ICM and TE grades are provided.(A) AA: Good graded ICM and TE. (B) AB: Good ICM with Average TE.(C) AC: Good ICM with Poor TE. (D) BA: Average ICM with Good TE. (E) BB: Average ICM and TE. (F) BC: Average ICM with Poor TE. (G) CA: Poor ICM with Good TE. (H) CB: Poor ICM with Average TE. (I) CC: Poor ICM and TE. [file peerj-10-13935-s003.png]
